# Websites recommended to patients with adolescent idiopathic scoliosis at first point of diagnosis: a content analysis

**DOI:** 10.1186/1748-7161-7-S1-O35

**Published:** 2012-01-27

**Authors:** S Wellburn, J Bettany-Saltikov, D Martin, P Van Schaik

**Affiliations:** 1 Teesside University, Middlesbrough, UK

## Purpose

To examine the content of websites suggested to Adolescent Idiopathic Scoliosis patients by clinicians at fourteen specialist UK scoliosis centres.

## Background

To support information provided during consultation health professionals commonly recommend other sources of information material. The use of electronic means to access health information is becoming more popular as the Internet becomes more widely available [1]. The problem is not in finding information but in assessing the validity and credibility of that information.

## Materials and methods

Eight websites, SAUK, BSS, SRS, BSRF, BASS, Eurospine, Medikidz and iScoliosis, identified from previous clinician survey responses at the scoliosis centres, were recommended to patients. These were analysed for content and relevance using the DISCERN instrument [2].

## Results

Information was found to be lacking on most websites, detailing how different treatments work, their benefits and risks and how these treatments may affect the quality of life of patients. The source of the information supplied on the websites and the date it was produced was rarely identified. Information regarding the signs, symptoms and aetiology of the condition was well presented.

## Conclusions

The websites that are recommended to patients should contain up to date evidence-based information that is impartial and written in plain language. They should also contain material that has been designed to meet the information needs of patients Healthcare professionals need to be fully aware of the content of the websites they recommend, to enable them to suggest the most appropriate information sources.

## References

[B1] Ullrich PF Vaccaro AR Patient education on the Internet: opportunities and pitfalls Spine 2002 27 E185 E188 10.1097/00007632-200204010-00019 11923675

[B2] Charnock D Sheppard S Needham G Gann R DISCERN: an instrument for judging the quality written consumer health information on treatment choices Journal of Epidemiology and Community Health 1999 53 105 111 10.1136/jech.53.2.105 10396471 PMC1756830

